# Inducible expression of immediate early genes is regulated through dynamic chromatin association by NF45/*ILF2* and NF90/NF110/*ILF3*

**DOI:** 10.1371/journal.pone.0216042

**Published:** 2019-04-25

**Authors:** Ting-Hsuan Wu, Lingfang Shi, Anson W. Lowe, Mark R. Nicolls, Peter N. Kao

**Affiliations:** 1 Pulmonary and Critical Care Medicine, Stanford University School of Medicine, Stanford, California, United States of America; 2 Biomedical Informatics, Stanford University School of Medicine, Stanford, California, United States of America; 3 Gastroenterology and Hepatology, Stanford University School of Medicine, Stanford, California, United States of America; University of Texas Rio Grande Valley, UNITED STATES

## Abstract

Immediate early gene (IEG) transcription is rapidly activated by diverse stimuli. This transcriptional regulation is assumed to involve constitutively expressed nuclear factors that are targets of signaling cascades initiated at the cell membrane. NF45 (encoded by *ILF2)* and its heterodimeric partner NF90/NF110 (encoded by *ILF3*) are chromatin-interacting proteins that are constitutively expressed and localized predominantly in the nucleus. Previously, NF90/NF110 chromatin immunoprecipitation followed by deep sequencing (ChIP-seq) in K562 erythroleukemia cells revealed its enriched association with chromatin at active promoters and strong enhancers. NF90/NF110 specifically occupied the promoters of IEGs. Here, ChIP in serum-starved HEK293 cells demonstrated that NF45 and NF90/NF110 pre-exist and specifically occupy the promoters of IEG transcription factors *EGR1*, *FOS* and *JUN*. Cellular stimulation with phorbol myristyl acetate increased NF90/NF110 chromatin association, while decreasing NF45 chromatin association at promoters of *EGR1*, *FOS* and *JUN*. In HEK293 cells stably transfected with doxycycline-inducible shRNA vectors targeting NF90/NF110 or NF45, doxycycline-mediated knockdown of NF90/NF110 or NF45 attenuated the inducible expression of *EGR1*, *FOS*, and *JUN* at the levels of transcription, RNA and protein. Dynamic chromatin association of NF45 and NF90/NF110 at IEG promoters are observed upon stimulation, and NF45 and NF90/NF110 contribute to inducible transcription of IEGs. NF45 and NF90/NF110 operate as chromatin regulators of the immediate early response.

## Introduction

The rapid cellular response that occurs upon recognition of biological or environmental signals is crucial for adaptation and survival of the organism [1–3]. The subset of genes that are rapidly expressed upon induction are termed immediate early genes (IEG) [4]. Inducible expression of IEGs in response to diverse regulatory signals underlies acute inflammation [5–8], neuronal activity [9], cell proliferation, and differentiation [1, 10, 11]. Aberrant expression of IEGs is involved in malignant cellular transformation [12] and is a feature of diverse cancers [13, 14].

Upon stimulation, initial expression of IEGs occurs on the timescale of minutes to hours [4, 15]. The earliest protein products of these IEGs critically include ‘forward-driving’ transcription factors such as *EGR1*, *FOS*, and *JUN*, followed by expression of delayed primary response genes (D-PRG), followed by protein synthesis-dependent expression of secondary response genes (SRG) [15]. The AP-1 components *FOS* and *JUN* regulate transcription of many cytokine genes and acute inflammation. The intensity and duration of signaling is attenuated through IEG induction of the family of dual-specificity phosphatases/ MAPK phosphatases [16].

Regulation of this hierarchical program upon cellular stimulation does not require *de novo* protein synthesis. Transcriptional regulation of IEGs is thus assumed to involve pre-existing nuclear factors that are constitutively expressed, which are targets of signaling cascades initiated at the cell membrane. Features of IEG promoters include over-representation of transcription factor binding sites and high affinity TATA boxes [4]. Chromatin structure of IEGs shows enrichment of active chromatin marks and “poised” accumulation of RNA polymerase II [15]. Stimulation-induced chromatin remodeling at promoters of IEGs exposes specific DNA binding sequences for transcription factors such as serum-response factor (SRF), nuclear factor kappa B (NF-kB), and cyclic AMP response element-binding protein (CREB) [4]. Transcription of DNA by RNA Polymerase II complex into RNA [17] is followed by post-transcriptional regulation at the levels of RNA splicing, nuclear export, stabilization, and translational regulation of the nascent transcripts [4].

Nuclear Factor 90 (NF90 and splice variant NF110, both encoded by the *ILF3* gene) and Nuclear Factor 45 (NF45, encoded by the *ILF2* gene) are multifunctional DNA- and RNA-binding proteins originally purified and cloned based on their inducible and specific DNA-binding to the nuclear factor of activated T-cells / antigen receptor response element-2 (NF-AT/ ARRE-2) sequence in the *IL2* promoter from activated Jurkat T-cells [18, 19]. NF90/NF110 and NF45 frequently interact as a heterodimer through their shared dimerization zinc-finger (DZF) domains [20]. NF90/NF110 and splice variant NF110 contain two dsRNA binding domains, and both NF90/NF110 and NF45 contain a single arginine/glycine/glycine (RGG) domain that is capable of binding to both DNA and RNA [21, 22]. The interactions of NF90/NF110 and NF45 with chromatin have been demonstrated at several regulatory regions in addition to *IL2* [23–25], including promoters of *FOS* [26], *PLAU* [27] and enhancer of HLA-DR alpha [28] and *IL13* [29]. Nakadai *et al*. used a combination of *in vitro* transcription and *in vivo* reporter gene assays established that NF45, NF90/NF110 operate as transcriptional coactivators of *FOS* [26].

NF90/NF110 and NF45 have been shown to regulate embryonic pluripotency [30], and development. NF90/NF110 is required for normal development. Mice with targeted disruption of NF90/NF110 were born small and weak and succumbed to perinatal death from neuromuscular respiratory failure [23]. NF45 knockout in mice resulted in early embryonic lethality (Zhao and Kao, unpublished results). NF45 physically interacts with Oct4 and Nanog in embryonic stem cells (ESC) to promote pluripotency [31]. Targeted disruption of NF90/NF110 and NF45 impaired ESC proliferation and promoted differentiation to an epiblast-like state [30]. NF90/NF110 and NF45 regulate cell cycle progression [21, 23, 32], cell growth and proliferation [32–38], and are amplified, overexpressed and mutated in diverse cancers [39, 40].

We recently characterized NF90/*ILF3* as a transcription factor involved in promoting proliferation and renewal over differentiation in K562 erythroleukemia cells using chromatin immunoprecipitation followed by deep sequencing (ChIP-seq) [41]. Rigorous statistical testing between biological replicates with Irreproducible Discovery Rate (IDR) analysis revealed chromatin occupancy of NF90/NF110 at 9,081 specific genomic sites, with over a third occurring at promoters of protein-coding genes.

Further analysis of NF90/NF110 chromatin occupancy in a context of histone modifications revealed enrichment of NF90/NF110 occupancy frequencies at active promoters and strong enhancers. To investigate the functional role of NF90/NF110 in transcriptional regulation in K562 cells, we compared the 2,927 genes with NF90/NF110 chromatin occupancy in its proximal promoter to a dataset of 446 genes that were differentially expressed upon NF90/NF110 knockdown by shRNA. In K562 cells grown in 10% serum at basal growth conditions, this integrated analysis of genes under transcription regulation by NF90/NF110 revealed an overrepresentation of IEGs.

Tullai *et al*. previously demonstrated that the cellular response to growth factor stimulation involves initial induction of IEGs, followed by delayed expression of primary response genes that also do not require initial protein synthesis, and finally, secondary response genes [15]. Here, we examined NF90/NF110 ChIP-seq data in K562 cells at normal growth conditions. Compared to delayed primary response genes or secondary response genes, we found enriched NF90/NF110 occupancy at promoters of IEGs, including ‘forward-driving’ transcription factors *EGR1*, *FOS*, and *JUN*.

The chromatin occupancy of NF90/NF110 at promoters of IEGs in normally growing K562 cells suggested to us that NF90/NF110, together with its frequent heterodimeric partner, NF45, might contribute to regulation of IEG expression. In this study, we test the hypothesis that NF90/NF110 and NF45 contribute to transcriptional regulation of IEG transcription factors *EGR1*, *FOS*, and *JUN*.

## Materials and methods

### Cell culture and stimulation

Human embryonic kidney (HEK) 293 cells (ATCC, Manassas, VA) were maintained in Dulbecco’s modified Eagle’s medium supplemented with 10% fetal calf serum with Penicillin and Streptomycin. Cells were serum starved for 12 h before stimulation with 20 ng/ml phorbol 12-myristate 13-acetate (PMA) for indicated durations, harvested by Trypsin-EDTA, centrifugation, and frozen at -80°C.

### Reagents

The following antibodies were used for chromatin immunoprecipitation and/or Western immunoblotting: NF90/NF110 (mouse mAb DRBP76; BD 612154), NF45 (mouse mAb NF45 H-4; Santa Cruz 365283), the large subunit of RNA polymerease II (mouse mAb 8WG16; Covance). Antibodies used for Western immunoblotting include: anti-EGR1 (rabbit mAb EGR1 15F7; Cell signaling 4153), anti-FOS (rabbit mAb FOS 9F6; Cell signaling 2250), anti-JUN (rabbit mAb JUN 60A8; Cell signaling 9165), and anti-GAPDH (rabbit pAb GAPDH; Abcam ab9485).

### Chromatin immunoprecipitation

Cells were harvested and crosslinked in 1% formaldehyde for 10 minutes at room temperature. Pellets were re-suspended in hypotonic buffer (20 mM HEPES, 10 mM KCl, 1mM EDTA, 10% glycerol) supplemented with Pierce Protease Inhibitor Tablets (Thermo Fisher), placed on ice for 10 minutes and centrifuged to remove cytoplasmic supernatant. Chromatin was released from nuclear pellet with mild chemical lysis with Radio-immunoprecipitation assay buffer (RIPA) containing 0.1% SDS and 1% Triton-X-100. Protein-bound chromatin was sheared with mechanical sonication (Branson 250 Sonifier) at 35% output with 20 second pulses for 30 cycles to obtain chromatin fragments of resolution at 200–500 bps. Successful chromatin fragmentation was assayed by reverse cross-linking and electrophoresis through 0.8% agarose gel to confirm enrichment of small chromatin fragments at the target range. 15% of chromatin fragments were designated as input, and the rest were pre-cleared with untreated protein A/G agarose beads (Santa Cruz), then incubated in protein A/G agarose beads that were pre-conjugated overnight at 4°C with monoclonal antibodies to NF90/NF110 (BD mAB DRBP76), NF45 (Santa Cruz sc-365283), or the large subunit of RNA polymerease II (8WG16; Covance). Input chromatin, as we as chromatin fragments enriched for NF90/NF110 or NF45-binding, were eluted in 1% SDS at 65°C for 15 minutes. Chromatin was reverse cross-linked from protein at 65°C overnight. RNase A (100 ug, Sigma Aldrich) was added to sample to incubate at 37°C for 30 minutes, and Proteinase K (100 ug, Invitrogen) was then added to sample to incubate at 45°C for 30 minutes. Input and ChIP DNA was purified using PCR purification column (Qiagen) per manufacturer protocol and eluted in 35 ul EB buffer (10 mM Tris-Cl, pH 8.5).

Input and ChIP DNA was used as template for polymerase chain reactions using primers amplifying specific genomic regions to interrogate chromatin occupancy by NF90/NF110, NF45, or RNA Polymerase II. PCR primers were synthesized by the Protein and Nucleic Acid Facility at Stanford University, and designed to amplify the *EGR1* proximal promoter: F 5’–TTCCCCAGCCTAGTTCACGCCTAGGAGCC– 3’, R 5’–ATATGGCATTTCCGGGTCGCAGCTGG– 3’; *EGR1* gene body: F 5’–GCAGTGGAGGGGGATTCTCCGTA– 3’, R 5’–CCGGCTACCATTGACTCCCGAG– 3’; *FOS* proximal promoter: F 5’–CCGCGAGCAGTTCCCGTCAATCCCTC– 3’, R 5’–GCAGTTCCTGTCTCAGAGGTCTCGTGGGC– 3’; *FOS* gene body: F 5’–CTCACGTCGGCTTTCCCCTTCT– 3’, R 5’–GGGACTCCGAAAGGGTGAGGG– 3’; *JUN* proximal promoter: F 5’–CCTCCCGGGTCCCTGCATCCCC– 3’, R 5’–ACGCCTCTCGGCCCTCTCTTCCC– 3’; *JUN* gene body: F 5’–ATGCCCTCAACGCCTCGTTCC– 3’, R 5’–CGAGGTGAGGAGGTCCGAGTTC– 3’; *HBB* locus control region: F 5’–CCTCGGCCTCCCAAAGTGCCAGGATTACAG– 3’, R 5’–ACAAGCATGCGTCACCATGCCTGGC– 3’; *HBB* gene body: F 5’–AGTCCAAGCTAGGCCCTTTTGCTAA– 3’, R 5’–GGCATTAGCCACACCAGCCAC– 3’. Quantitative PCR was performed using Applied Biosystems StepOne instrument and SybrGreen detection.

### Molecular cloning and transfection

The pINDUCER lentiviral toolkit for inducible RNA interference [42] was utilized for inducible shRNA knockdown of NF90/NF110 or NF45. pInducer10-mir-RUP-PheS was a gift from Stephen Elledge (Addgene plasmid # 44011), containing a constitutively expressed transcript encoding the puromycin resistance gene, and a doxycycline-inducible cassette that expresses target shRNA sequence as well as turboRFP fluorescent protein. Validated shRNA sequences D2 directed against NF90/NF110/NF110 (5’–GUGCUGGUUCCAACAAAA– 3’) and D5 directed against NF45 (5’–AGUCGUGGAAAGCCUAAGA– 3’) were designed as described by Guan *et al*. [19] and sub-cloned into the doxycycline-inducible cassette driven by the tetracycline-responsive TRE2 promoter in pINDUCER10 to create plasmids pINDUCER10-shNF90/NF110 (D2) and pINDUCER10-shNF45 (D5).

HEK293 cells at 70% confluency were transfected with either pINDUCER10-shNF90/NF110 (D2) or pINDUCER10-shNF45 (D5) using JetPrime transfection reagent (Polyplus). Beginning at 48 h after transfection, drug selection for stable transfection was imposed using puromycin (1 μg/ml).

HEK293 cells stably expressing D2 or D5 were treated without or with doxycycline (1 ug/ul) in DMEM medium supplemented with 10% FBS for 96 h to achieve maximal attenuation of NF90/NF110 or NF45 expression. Cells were then serum-starved for 12 h with continuing presence of doxycycline, before stimulation with PMA for the indicated durations.

### Reverse transcription polymerase chain reaction

RNA levels were assayed using reverse transcription PCR. Total RNA was isolated from cells using the RNeasy mini kit (Qiagen). First-strand cDNA was synthesized from 2 ug of total RNA using Superscript IV VILO reverse transcriptase (Invitrogen) with a mix of oligo(dT) and random hexamer primers per manufacturer protocol, which was used as template for polymerase chain reactions. Primers were designed to amplify: *EGR1* mRNA: F 5’–GCACCTGACCGCAGAGTCTTTTCCT– 3’, R 5’–GGTGTTGCCACTGTTGGGTGCAG– 3’; *EGR1* pre-mRNA: F 5’–CTCTGCCACTGGTGCGGGTC– 3’, R 5’–GGTGTTGCCACTGTTGGGTGCAG– 3’; *FOS* mRNA: F 5’–CTGTCAACGCGCAGGACTTCTGC– 3’, R 5’–GCTCGGCCTCCTGTCATGGTCT– 3’; *FOS* pre-mRNA: F 5’–CATGCGGCACTGGGAACTCGC– 3’, R 5’–GCTCGGCCTCCTGTCATGGTCT– 3’; *JUN* mRNA: F 5’–CGATGCCCTCAACGCCTCGTTC– 3’, R 5’–GTGATGTGCCCGTTGCTGGACTG– 3’; *ACTB* mRNA: F 5’–CCAATCAGCGTGCGCCGTTCC– 3’, R 5’–ATCATCCATGGTGAGCTGGCGG– 3’; *ACTB* pre-mRNA: F 5’–GGCAAGGGCGCTTTCTCTGCAC– 3’, R 5’–ACATAGGAATCCTTCTGACCCATGCCC– 3’. Quantitative PCR was performed using Applied Biosystems StepOne instrument and SybrGreen detection. Transcript abundance was normalized to *ACTB*.

### Western immunoblotting

Cells were harvested and whole cell extracts were prepared by incubation on ice for 30 minutes in 8M urea lysis buffer (8M urea, 300 mM NaCl, 0.5% NP-40, 50 mM Na_2_HPO_4_, 50 mM Tris-HCl, 1 mM PMSF) supplemented with Pierce Protease Inhibitor Tablets (Thermo Fisher). Twenty micrograms of protein were separated by SDS-PAGE and transferred to polyvinylidene difluoride (PVDF) membranes. Primary antibodies were used at 1:1000 dilution, followed by secondary antibodies (anti-mouse or anti-rabbit horseradish peroxidase-coupled, Santa Cruz) at 1:10,000 dilution, and signals were detected with enhanced chemiluminescence (Amersham).

### Immunofluorescence staining

Cells were passaged and seeded on chamber slides. Upon serum starvation and stimulation, cells were fixed and permeabilized in 100% methanol at room temperature for 15 minutes, and bleaching of native fluorescence was confirmed with microscopy. After rinsing three times with PBS, cells were blocked in 5% FBS/ 0.3% Triton X-100 in PBS for 1 h at room temperature. The chamber slides were then incubated with anti-NF90/NF110/NF110 (mouse mAb BD, 1:200) or anti-NF45 (mouse mAb Santa Cruz, 1:200); and anti-EGR1 (rabbit mAb Cell Signaling, 1:100), anti-FOS (rabbit mAb Cell Signaling, 1:100), or anti-JUN (rabbit mAb Cell Signaling, 1:100) diluted in 0.5% FBS/ 0.03% Triton X-100 in PBS overnight at 4°C. After overnight incubation, unbound primary antibody was removed by washing the slides three times in PBS. Fluorescent conjugated secondary antibodies anti-mouse (Goat anti-Mouse IgG Alexa Fluor 488 Invitrogen, 1:500) and anti-rabbit (Goat anti-Rabbit IgG Alexa Fluor 594 Invitrogen, 1:500) were applied to slides for 1 h at room temperature protected from light. Slides were washed three times with PBS, counterstained with DAPI nuclear stain (Pierce, 1 mg/ml), and mounted using VECTASHIELD antifade solution (Vector Laboratories). Imaging was performed on a Zeiss confocal laser scanning microscope (LSM 880, Zeiss) with a 20X objective lens.

## Results

### Enriched NF90/NF110 chromatin occupancy at promoters of immediate early genes

In collaboration with the ENCODE consortium we performed ChIP-seq for NF90/NF110 in K562 erythroleukemia cells (ENCODE ENCSR632TJQ) and discovered chromatin occupancy at over 9000 specific sites [41]. Increased NF90/NF110 chromatin occupancy occurred at active promoters and strong enhancers, and NF90/NF110 clustered with transcription factors exhibiting preference for promoters over enhancers (*POLR2A*, *MYC*, *YY1)*. Integrating differential gene expression analysis following shRNA knockdown of NF90/NF110 with ChIP-seq data in K562 cells lead us to conclude that NF90/NF110 operates as a hierarchical transcription factor to promote cell proliferation over differentiation.

**Fig 1:**
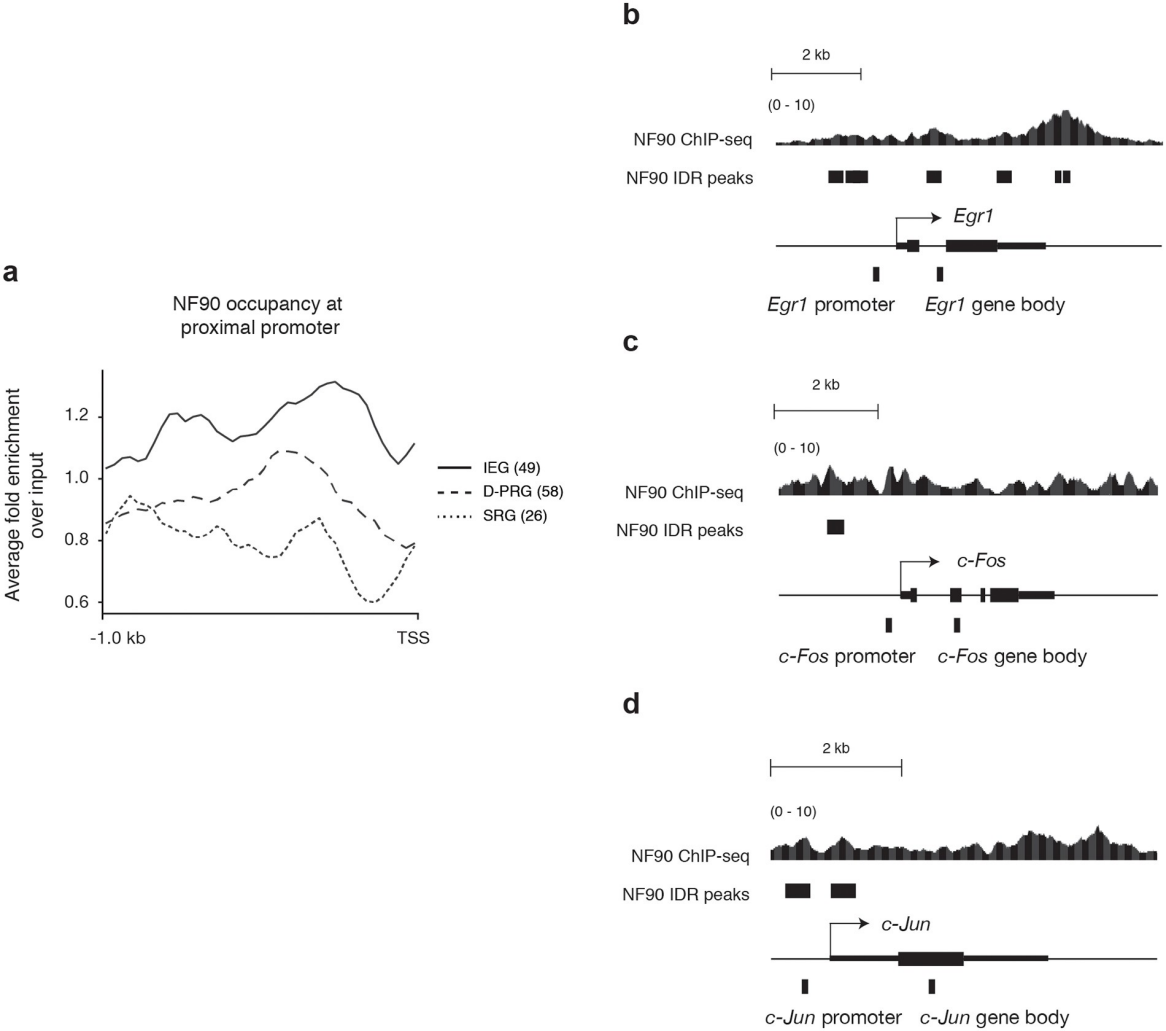
NF90/NF110 occupancy at promoters of immediate early genes in K562 cells. (a) Average occupancy profile of NF90/NF110/NF110 at proximal promoters within 1000 bp upstream of transcription start site (TSS) of 49 immediate early genes (IEG), 58 delayed primary response genes (D-PRG), and 26 secondary response gene (SRG) defined by Tullai et al. *x*-axis: Relative position near TSS. *y*-axis: Fold-change over input of NF90/NF110/NF110 ChIP-Seq signal. (b-d) Signal tracks of NF90/NF110 chromatin occupancy determined by ChIP-seq at promoters of IEGs *EGR1*, *FOS*, and *JUN*; retrieved from UCSC genome browser [41]. NF90/NF110 ChIP-seq signal (fold change over input) is presented, aligned to peaks called by Irreproducible Discovery Rate (IDR) analysis that measures consistency between replicates. Relative position of the amplicons in the proximal promoter and gene body of each gene used for subsequent ChIP-PCR experiments are indicated.

Here, to characterize how NF90/NF110 might positively regulate proliferation of K652 cells, we determined the average chromatin occupancy profile of NF90/NF110 at the proximal promoters of IEGs, D-PRGs, and SRGs (Fig 1a). The specific genes in each group and the genomic coordinates of the proximal promoters are tabulated (S1 Table). NF90/NF110 chromatin occupancy frequency was highest at the proximal promoter of IEGs, followed by D-PRGs, then SRGs. There was approximately a two-fold enrichment in NF90/NF110/NF110 occupancy frequency at the proximal promoter of IEGs compared to SRGs.

Here, we focus on NF90/NF110 chromatin occupancy at IEGs that are ‘forward-driving’ transcription factors: *EGR1*, *FOS*, and *JUN*. We sought to determine whether NF90/NF110, and its heterodimeric partner NF45, may hierarchically regulate transcription factors that control the immediate early responses of cells to diverse stimuli. NF90/NF110 chromatin occupancy frequency (fold-change over input) is graphed as a continuous variable, and Irreproducible Discovery Rate (IDR) analysis marks the discrete genomic intervals where rigorous statistical testing between biological replicates indicates strong confidence in NF90/NF110 chromatin occupancy (Fig 1b–1d). At the *EGR1*, *FOS* and *JUN* loci, continuous NF90/NF110 chromatin occupancy is found at the transcription start site (TSS), throughout the gene body, and at the transcription end site (TES), consistent with previous findings [41]. Most significantly, we demonstrate NF90/NF110 chromatin occupancy at proximal promoters of immediate early transcription factors *EGR1*, *FOS*, and *JUN* (Fig 1b–1d).

### Dynamic chromatin association of NF90/NF110 and NF45 at promoters of immediate early genes during cell stimulation

To generalize our finding that NF90/NF110 occupied the promoters of immediate early transcription factors in normal growing K562 cells, we extended our study of IEG regulation to the human embryonic kidney (HEK) 293 cell line, which demonstrates no tissue-specific gene expression signature and a transformed immortal phenotype [43], and is amenable to stable cell transfections for cell biology studies.

Based on the bioinformatics results that demonstrated enrichment of NF90/NF110 occupancy at promoters at immediate early transcription factors, and the frequent dimerization interaction between NF90/NF110 and NF45 mediated by their shared domain associated with zinc fingers (DZF), we hypothesized that, upon cellular stimulation, NF90/NF110 and NF45 might coordinately regulate expression of immediate early transcription factors *EGR1*, *FOS*, and *JUN*. The stimulation of choice for these experiments was phorbol 12-myristate 13-acetate (PMA), an activator of protein kinase C (PKC), previously demonstrated to stimulate inducible binding of NF90/NF110 to the *IL2* promoter [23, 24], and known to rapidly induce expression of IEGs, including *EGR1*, *FOS*, and *JUN* [7]. For maximal contrast of IEG induction, HEK293 cells were serum starved overnight. Quiescent cells were then stimulated with 20 ng/ml PMA to achieve rapid induction of IEGs.

We performed ChIP-PCR using monoclonal antibodies to NF90/NF110 or NF45, and interrogated amplicons within 500 bp upstream of the transcription start sites of *EGR1*, *FOS*, and *JUN* selected based upon the continuous NF90/NF110 chromatin occupancy frequency in K562 cells (Fig 1b–1d). As a negative control amplicon we selected a region of the human beta globin locus (*HBB*) that exhibited low NF90/NF110 chromatin occupancy frequency in K562 cells.

**Fig 2:**
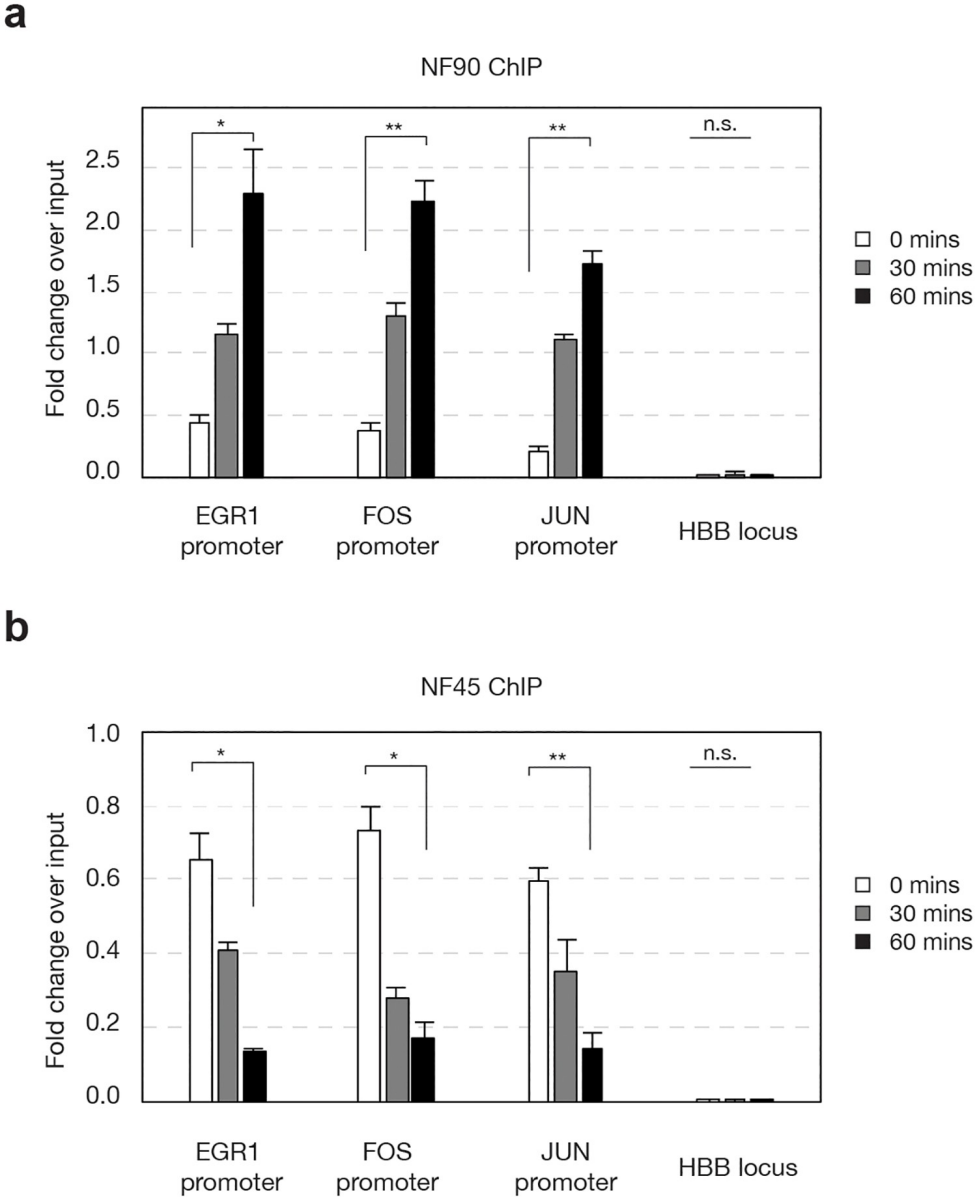
Dynamic associations of NF90/NF110 and NF45 with promoters of IEGs upon stimulation. HEK293 cells were serum starved for 24 h, then stimulated by PMA (20 ng/ml) for indicated durations. Chromatin immunoprecipitation was performed with antibodies against NF90/NF110 (a) or NF45 (b). Abundance of IEG promoter fragments were assessed by quantitative polymerase chain reaction for input sheared chromatin, or specific immunoprecipitates. *N* = 3 biological replicates; One-way analysis of variance (ANOVA) test followed by post-hoc Student’s *t*-test corrected by Bonferroni; data reported as mean ±s.e.m of fold-change over input, * *P* <0.05, ** *P* < 0.01.

NF90/NF110 chromatin occupancy at proximal promoters of *EGR1*, *FOS*, and *JUN* in nonstimulated HEK293 cells was present at low basal levels (Fig 2a, 0 min, open bars). Stimulation with PMA triggered increases in NF90/NF110 chromatin occupancy at the *EGR1*, *FOS*, and *JUN* promoters at 30 min, and a further significant increase at 60 min (Fig 2a black bars, *P* < 0.05).

NF45 chromatin occupancy at proximal promoters of *EGR1*, *FOS*, and *JUN* in nonstimulated HEK293 cells was present at substantial levels (Fig 2b, 0 min, open bars), in contrast to NF90/NF110. Unexpectedly, stimulation with PMA triggered decreases in NF45 chromatin occupancy at the *EGR1*, *FOS*, and *JUN* promoters at 30 min, and further significant decrease at 60 min (Fig 2b, black bars, *P* < 0.05).

NF45 and NF90/NF110 copurify, and frequently interact as heterodimers through their shared DZF domains [18–20]. Our discovery of dynamic and reciprocal chromatin associations by NF90/NF110 and NF45 during cell stimulation suggest that each protein contributes distinctly and non-redundantly to transcriptional activation.

### Establishment and characterization of stable HEK293 cells with doxycycline-regulated RNAi targeting NF90/NF110 and NF45

The chromatin occupancy of NF90/NF110 and NF45 at the proximal promoters of *EGR1*, *FOS*, and *JUN* supports our hypothesis that NF90/NF110 and NF45 hierarchically regulate transcriptional activation of ‘forward-driving’ IE transcription factors. To study the functional roles of NF90/NF110 and NF45 in regulating *EGR1*, *FOS*, and *JUN*, we employed RNA interference using validated [21] shRNA sequences against NF90/NF110 (D2) or NF45 (D5) to knockdown NF90/NF110 or NF45 proteins, and examined the consequences upon inducible expression of *EGR1*, *FOS*, and *JUN*.

Because NF90/NF110 and NF45 are genes essential for normal development, as well as for cellular growth and proliferation, we anticipated that constitutive knockdown in HEK293 cells might adversely affect cell viability, as previously reported [21]. Therefore, we established a system for doxycycline-regulated RNAi knockdown of NF90/NF110 and NF45. The pINDUCER vectors are multicistronic plasmids in which a strong ubiquitin promoter drives constitutive expression of reverse tetracycline transactivator protein and resistance to puromycin, and a tetracycline-regulated promoter drives expression of turboRed fluorescent protein and shRNA [42]. pINDUCER 10 plasmids were created with shRNA sequences against NF90/NF110 (D2) or NF45 (D5). We transfected HEK293 cells with either pINDUCER-shNF90/NF110 (D2) or pINDUCER-shNF45 (D5), and applied puromycin selection to generate stably-transfected HEK293 cells with doxycycline-regulated shRNAs directed to NF90/NF110 (D2) or NF45 (D5).

**Fig 3:**
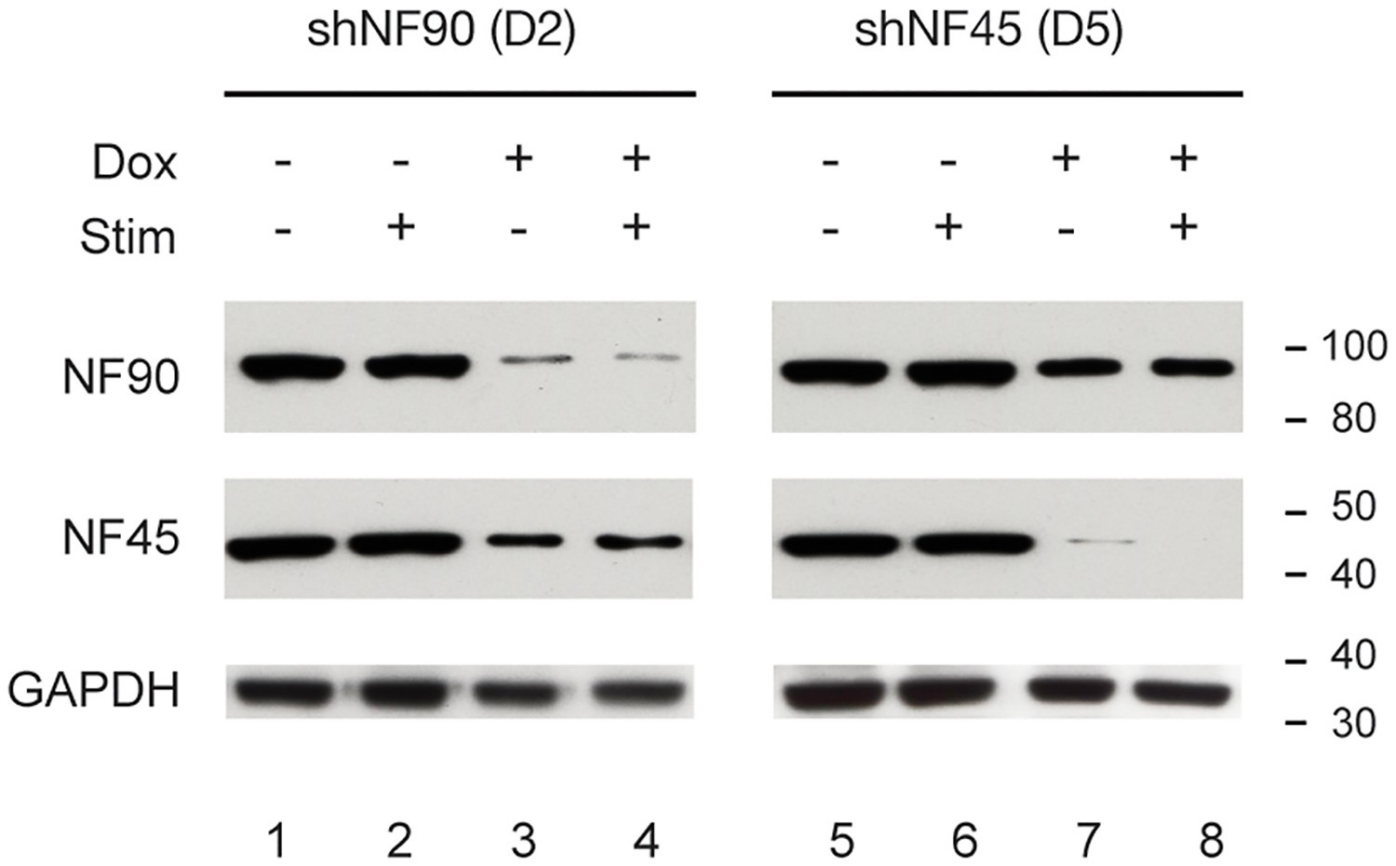
Doxycycline-induced shRNAs specifically attenuated expression of NF90/NF110 or NF45 proteins. HEK293 cells stably transfected with pINDUCER10-shNF90/NF110 (D2) or pINDUCER10-shNF45 (D5) were treated without or with doxycycline for 96 h, then serum starved for 12 h and treated with PMA (20 ng/ml) for 2 h. NF90/NF110 or NF45 protein expression was detected by immunoblotting.

The efficacy of doxycycline-regulated knockdown of NF90/NF110 or NF45 in stably-transfected HEK293 D2 and D5 cells was characterized by Western immunoblotting (Fig 3). HEK293 cells were untreated (Dox-), or treated with 1 μg/ml doxycycline (Dox+), and whole cell lysates prepared with urea to achieve maximal extraction of these proteins from chromatin. Compared to Dox-, Dox+ HEK293 D2 cells (shRNA targeting NF90/NF110) demonstrated substantial reduction in NF90/NF110 protein expression (Fig 3, NF90/NF110: lanes 3, 4 vs. 1, 2). Additionally, compared to Dox-, Dox+ HEK293 D2 cells showed modest reduction in NF45 protein expression (Fig 3, NF45: lanes 3,4 vs. 1,2). Compared to Dox-, Dox+ treated HEK293 D5 cells (shRNA targeting NF45) demonstrated substantial reduction in NF45 protein expression (Fig 3, NF45: lanes 7,8 vs. 5,6). Additionally, compared to Dox-, Dox+ HEK293 D5 cells showed modest reduction in NF90/NF110 protein expression (Fig 3, NF90/NF110: lanes 7,8 vs. 5,6). Our results confirm that doxycycline effectively induced shRNA-mediated knockdown of NF90 or NF45 proteins in our D2 or D5 stable cell lines, respectively. Furthermore, the observed modest attenuation of the levels of NF45 or NF90/NF110 when the heterodimeric partner is targeted by RNAi is consistent with the previous suggestion that NF90/NF110 and NF45 interact and confer mutual stabilization [21].

### NF90/NF110 and NF45 positively regulate inducible transcription of immediate early genes upon cell stimulation

**Fig 4:**
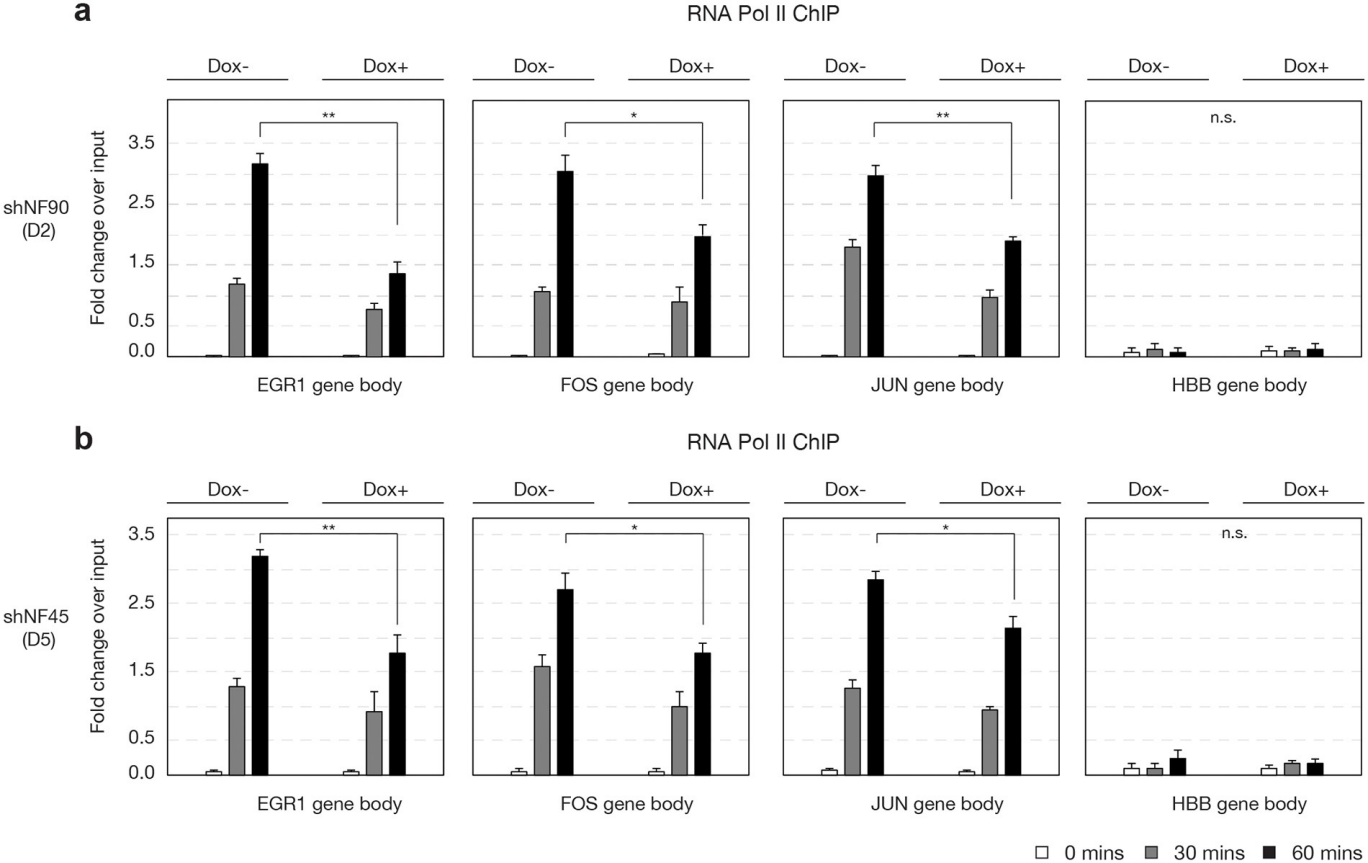
Reduced NF90/NF110 or NF45 expression attenuated inducible transcription of IEGs. HEK293 D2 or D5 cells were treated without or with doxycycline, then serum-starved for 12 h and treated with PMA (20 ng/ml) for 0, 30, or 60 min. ChIP was performed with antibody against RNA Polymerase II, and real time transcription of *EGR1*, *FOS*, *JUN* and *HBB* gene bodies was measured by qPCR. *N* = 3 biological replicates; One-way analysis of variance (ANOVA) test followed by post-hoc Student’s *t*-test corrected by Bonferroni; data reported as mean ± s.e.m of fold-change over input, * *P* < 0.05, ** *P*< 0.01.

To investigate whether knockdown of NF90 or NF45 attenuated transcription of IEGs, we performed RNA Pol II ChIP and used amplicons within the gene bodies of *EGR1*, *FOS*, and *JUN* (Fig 1b, 1c and 1d) as a measure of real-time transcription, as previously described [44]. In HEK293 cells stably expressing D2 (doxycycline inducible-shNF90) or D5 (doxycycline inducible-shNF45), we detected substantial increase in real-time transcription of *EGR1*, *FOS*, and *JUN* at 30 and 60 min upon PMA stimulation, compared to HBB (Fig 4a and 1b). Compared to Dox-, HEK293 D2 Dox+ cells exhibited significant reductions in transcription of *EGR1*, *FOS*, and *JUN* at 60 min (Fig 4a, black bars, *P* < 0.05). Similarly, compared to Dox-, HEK293 D5 Dox+ cells exhibited significant reductions in transcription of *EGR1*, *FOS*, and *JUN* at 60 min (Fig 4b, black bars, *P* < 0.05).

**Fig 5:**
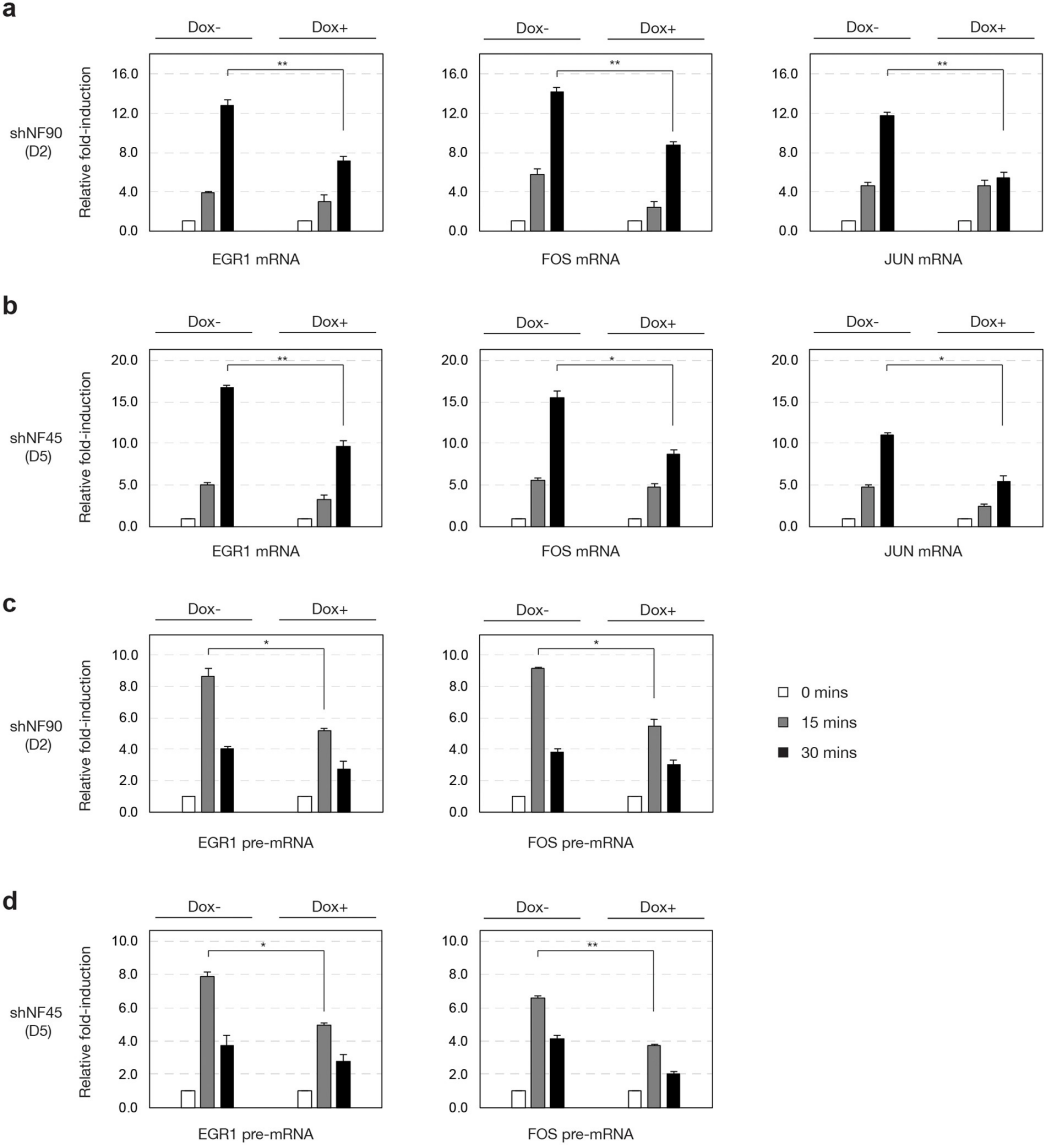
Reduced NF90/NF110 or NF45 expression attenuated inducible expression of IEG RNAs. HEK293 D2 or D5 cells were treated without or with doxycycline, then serum-starved for 12 h and treated with PMA (20 ng/ml) for 0, 15, or 30 min. Total RNA was prepared and used for reverse transcription qPCR to detect *EGR1*, *FOS*, and *JUN* mRNAs and pre-mRNAs. *ACTB* was used for normalization. *N* = 3 biological replicates; One-way analysis of variance (ANOVA) test followed by post-hoc Student’s *t*-test corrected by Bonferroni; data reported as mean ± s.e.m of fold-induction compared to 0 min, * *P* < 0.05, ** *P* < 0.01.

To understand the effect of NF90 and NF45 knockdown on the level of mature mRNA transcripts of IEGs upon cell stimulation, we performed reverse transcription PCR of *EGR1* and *FOS* using primer pairs that span at least one exon-exon junction to selectively detect the spliced mRNA, but not pre-mRNA. We also designed primers to amplify the intronless *JUN* transcript. In serum-starved HEK293 cells stably expressing D2 (doxycycline inducible-shNF90/NF110) mRNA expression of *EGR1*, *FOS*, and *JUN* is rapidly inducible by 15 min, and further increased at 30 min upon PMA stimulation (Fig 5a, Dox- panels). In doxycycline-treated D2 cells, the PMA-stimulated induction of *EGR1*, *FOS*, and *JUN* mRNA was attenuated compared to Dox- D2 cells (Fig 5a, black bars, *P* < 0.05).

We also characterized serum-starved HEK293 cells stably expressing D5 (doxycycline inducible-shNF45) for IEG mRNA expression. In Dox- D5 cells, stimulation with PMA rapidly induced mRNA expression of *EGR1*, *FOS*, and *JUN* in a pattern similar to Dox- D2 cells (Fig 5b, Dox- panels). In Dox+ D5 cells, PMA-stimulated induction of *EGR1*, *FOS*, and *JUN* expression was attenuated compared to Dox- D5 cells (Fig 5b, black bars, *P* < 0.05).

To investigate how reduced NF90/NF110 or NF45 expression may affect the earliest IEG RNA transcripts. Reverse transcription PCR primer pairs were designed to span intron-exon junctions of *EGR1* and *FOS* to detect pre-mRNAs that have not yet been spliced. In Dox- D2 cells, PMA-stimulated induction of *EGR1* and *FOS* pre-mRNA reached greatest levels at 15 min, followed by a decrease at 30 min (Fig 5c, Dox- panels). Thus, peak pre-mRNA expression was detected earlier than mature mRNA expression. Upon treatment of D2 cells with doxycycline, this PMA-stimulation expression of *EGR1* and *FOS* pre-mRNA was attenuated (Fig 5c, grey bars, *P* < 0.05). Characterization of Dox- D5 cells revealed similar patterns of *EGR1* and *FOS* pre-mRNA induction by PMA (Fig 5d, Dox- panels), and this was attenuated in Dox+ D5 cells (Fig 5d, grey bars, *P* < 0.05).

Taken together, our RNA pol II ChIP and reverse transcription PCR results establish that NF90/NF110 and NF45 both contribute positively to the inducible transcription of IEGs upon cell stimulation with PMA.

**Fig 6:**
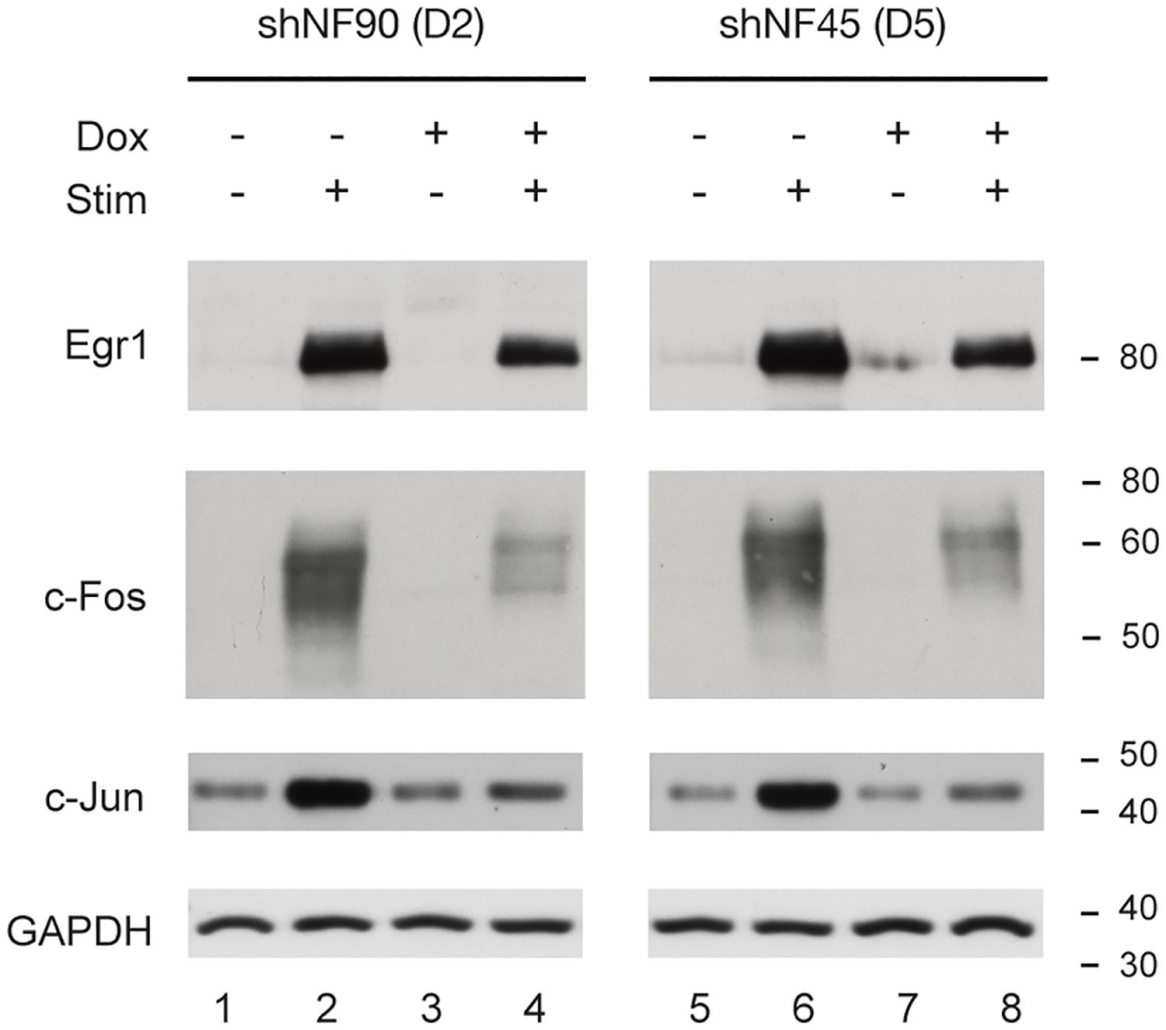
Reduced NF90/NF110 or NF45 expression attenuated inducible expression of IEG proteins. HEK293 D2 or D5 cells were treated without or with doxycycline, then serum starved for 12 h and treated with PMA (20 ng/ml) for 0, 2 h. Induction of IEGs *EGR1*, *FOS* and *JUN* were assessed by immunoblotting. GAPDH was used as a loading control.

To assay the inducible expression of IEGs at the protein level, we stimulated HEK293 D2 or D5 cells with PMA for 2 h and prepared whole cell lysates with urea to quantify by immunoblotting stimulated protein levels of *EGR1*, *FOS*, and *JUN*. We used Glyceraldehyde 3-phosphate dehydrogenase (GAPDH) as an internal control (Fig 6). HEK293 cells stably expressing shNF90/NF110 (D2) or shNF45 (D5) were cultured without or with doxycycline then serum-starved for 12h, followed by stimulation with PMA for 2 h. We determined that doxycycline-mediated knockdown of NF90/NF110 or NF45 proteins each attenuated the PMA-inducible protein expression of immediate early transcription factor proteins *EGR1*, *FOS*, and *JUN* (Fig 6, lane 4 vs. 2 and lane 8 vs. 6).

**Fig 7:**
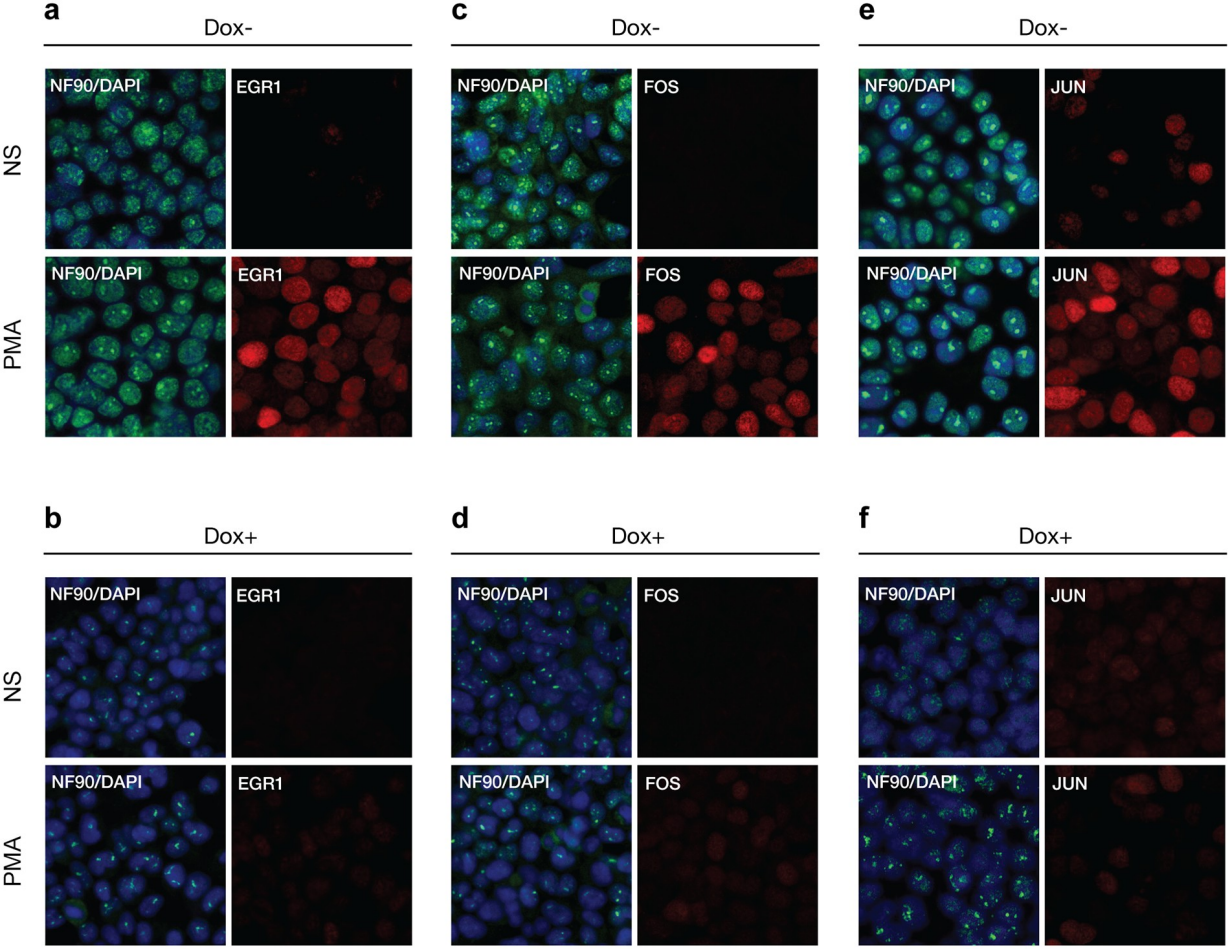
Reduced NF90/NF110 expression attenuated inducible expression IEGs by immunofluorescence analysis. HEK293 cells stably transfected with pINDUCER10-shNF90/NF110 (D2) or pINDUCER10-shNF45 (D5) were treated without or with doxycycline for 96 h, then seeded on chamber slides, and serum starved for 24 h and stimulated by PMA (20 ng/ml) for 2 h for maximal protein expression of IEG. Slides were incubated with mouse monoclonal antibodies against NF90/NF110, and rabbit monoclonal antibodies against *EGR1* (a-b), *FOS* (c-d), or *JUN* (e-f), then incubated with fluorescent conjugated secondary antibodies anti-mouse Alexa Fluor 488 and anti-rabbit Alexa Fluor 594. Slides were counterstained with DAPI, then visualized by confocal fluorescent microscopy. Panels (b,d,f) show doxycycline-mediated shRNA knockdown compared to (a,c,e) respectively.

**Fig 8:**
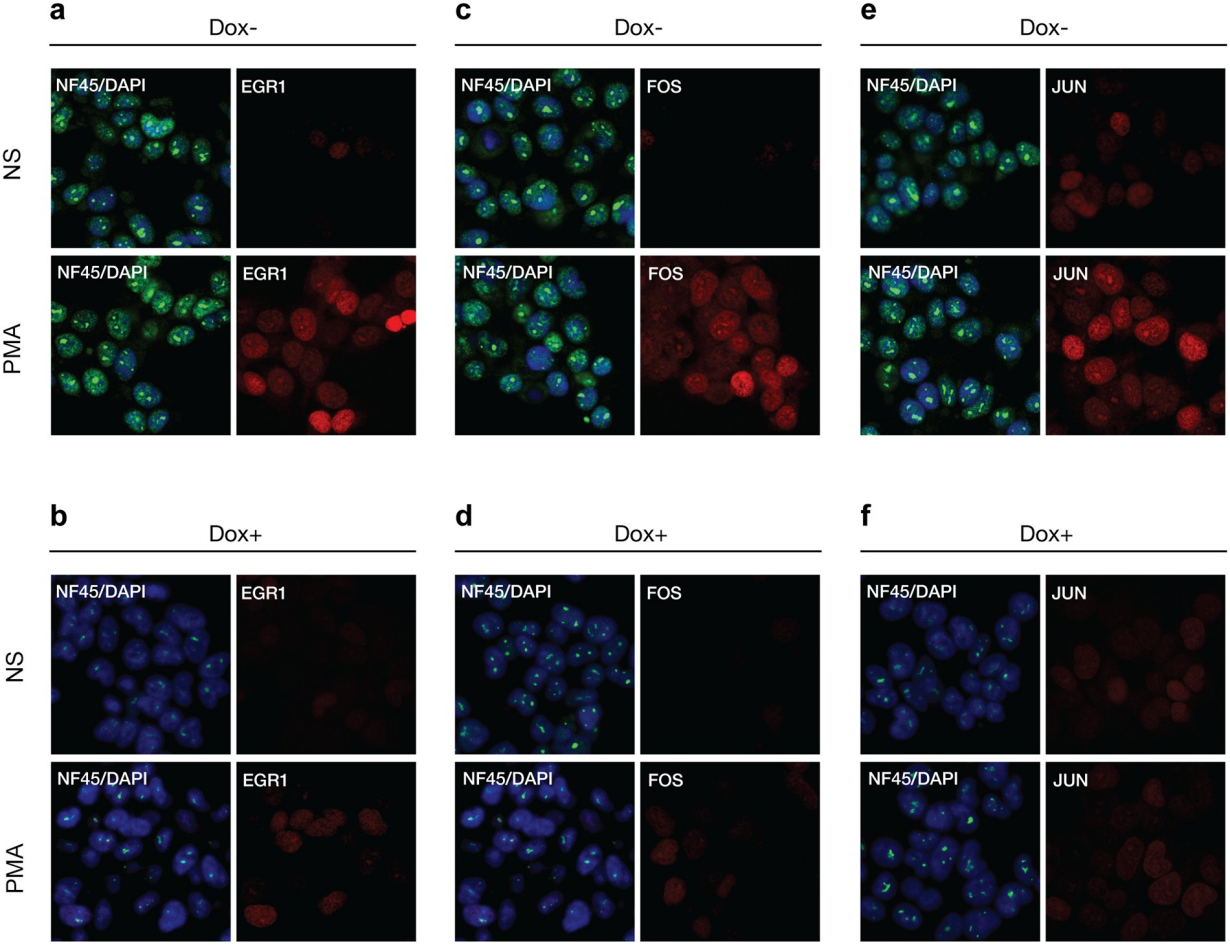
Reduced NF45 expression attenuated inducible expression IEGs by immunofluorescence analysis. HEK293 cells stably transfected with pINDUCER10-shNF90/NF110 (D2) or pINDUCER10-shNF45 (D5) were treated without or with doxycycline for 96 h, then seeded on chamber slides, and serum starved for 24 h and stimulated by PMA (20 ng/ml) for 2 h for maximal protein expression of IEG. Slides were incubated with mouse monoclonal antibodies against NF45, and rabbit monoclonal antibodies against *EGR1* (a-b), *FOS* (c-d), or *JUN* (e-f), then incubated with fluorescent conjugated secondary antibodies anti-mouse Alexa Fluor 488 and anti-rabbit Alexa Fluor 594. Slides were counterstained with DAPI, then visualized by confocal fluorescent microscopy. Panels (b,d,f) show doxycycline-mediated shRNA knockdown compared to (a,c,e) respectively.

To complement these population studies on IEG RNA and protein expression we performed immunofluorescence microscopy on HEK293 D2 and D5 cells for single cell characterizations of NF90/NF110 and NF45 regulation of PMA-inducible expression of *EGR1*, *FOS*, and *JUN* (Figs 7 and 8). Multiplexed immunofluorescence was achieved using mouse monoclonal antibodies to detect NF90/NF110 or NF45 proteins, and rabbit monoclonal antibodies to detect *EGR1*, *FOS*, and *JUN* proteins.

HEK293 cells stably expressing doxycycline-regulated shN90 (D2) were cultured on glass chamber slides without or with doxycycline before serum starvation overnight followed by stimulation with PMA for 2 h. Cells were fixed with methanol, membranes were permeabilized with Triton X-100 for incubation with primary mouse monoclonal antibody to NF90/NF110 together with rabbit monoclonal antibodies to *EGR1*, *FOS*, or *JUN*. All microscope exposure times for a given detection channel wavelength were identical across cell stimulation conditions (Fig 7). The NF90/NF110 immunoreactivity overlapped with DAPI staining, consistent with nuclear localization of NF90/NF110; there was punctate staining within the nuclei consistent with prior reports of NF90/NF110 interactions with nucleoli [45–48]. In D2 cells treated with doxycycline (Dox+), the immunofluorescence intensity of NF90/NF110 was substantially reduced compared to cells not exposed to doxycycline (Dox-) (Fig 7b, 7d, 7f vs. 7a, 7c and 7e). Compared to Dox–nonstimulated (NS) D2 cells, Dox–D2 cells stimulated with PMA demonstrated clear induction of *EGR1*, *FOS*, or *JUN* proteins within nuclei (Fig 7a, 7c, 7e; compare PMA vs. NS). In contrast to Dox–D2 cells (Fig 7a, 7c and 7e), Dox+ D2 cells exhibited marked attenuation of PMA-induced expression of *EGR1*, *FOS*, or *JUN* proteins (Fig 7b, 7d and 7f; compare PMA vs. NS).

Similarly, HEK293 cells stably expressing doxycycline-regulated shN45 (D5) were visualized using immunofluorescence microscopy (Fig 8). The NF45 immunoreactivity overlapped with DAPI staining, consistent with nuclear localization of NF45; there was punctate staining within the nuclei suggestive of NF45 interactions with nucleoli. Compared to Dox–D5 cells (Fig 8a, 8c and 8e), cells treated with doxycycline for 96 h (Dox+) demonstrated substantial attenuation of NF45 expression (Fig 8b, 8d and 8f). Stimulation of serum-starved Dox–D5 cells with PMA for 2 h induced substantial expression of *EGR1*, *FOS*, or *JUN* (Fig 8a, 8c and 8e; compare PMA vs. NS) proteins in nuclei. In contrast, stimulation of Dox+ D5 cells with PMA for 2 h showed marked attenuation of PMA-induced expression of *EGR1*, *FOS*, or *JUN* proteins (Fig 8b, 8d and 8f; compare PMA vs. NS).

These results from immunofluorescence microscopy demonstrate in single cells that knockdowns of NF90/NF110 or NF45 proteins are associated with attenuation of PMA-induction of immediate early transcription factors, *EGR1*, *FOS*, and *JUN*.

Taken together, we demonstrate dynamic chromatin occupancy by NF90/NF110 and NF45 at the proximal promoters of *EGR1*, *FOS*, and *JUN*. RNAi mediated knockdown of NF90/NF110 or NF45 specifically attenuates PMA-inducible transcription, RNA and protein expression of immediate early transcription factors *EGR1*, *FOS*, or *JUN*. We propose that NF90/NF110 and NF45 are chromatin regulators of IEG transcription.

## Discussion

We demonstrate that NF45 and NF90/NF110 exhibit dynamic chromatin association at the proximal promoters of the ‘forward-driving’ IEG transcription factors, *EGR1*, *FOS* and *JUN* in HEK293 cells. Genetic attenuation of NF45 or NF90/NF110 reduced PMA-stimulated transcription of *EGR1*, *FOS* and *JUN*. Our findings confirm and extend those of Nakadai *et al*. characterizing NF45 and NF90/NF110 as transcriptional coactivators of *FOS*. Operating as hierarchical transcriptional regulators of ‘forward-driving’ IE transcription factors, *EGR1*, *FOS* and *JUN*, NF45 and NF90/NF110 represent novel targets for regulation of acute inflammation, neuronal activity, cell proliferation, and differentiation. Increased expression of IEGs in malignant cellular transformation may be a consequence of overexpression of NF45 and/or NF90/NF110.

NF45 chromatin association was prominently detected at the proximal promoters of *EGR1*, *FOS* and *JUN* in serum-starved HEK293 cells prior to stimulation. Upon PMA stimulation, NF45 chromatin occupancy decreased at 30 and 60 min. In contrast, NF90/NF110 exhibited modest chromatin occupancy at the proximal promoters of *EGR1*, *FOS* and *JUN* in non-stimulated cells HEK 293 cells. Upon PMA stimulation, NF90/NF110 chromatin association increased at 30 and 60 min. These dynamic changes in NF45 and NF90/NF110 chromatin association are consistent with regulatory roles of NF45 and NF90/NF110 in IEG transcriptional activation stimulated by PMA.

NF45 and NF90/NF110 both contribute positively to expression of *EGR1*, *FOS* and *JUN*. Doxycycline-regulated RNAi knockdown of NF45 or NF90/NF110 significantly attenuated PMA-inducible expression of *EGR1*, *FOS* and *JUN* at the levels of transcription, RNA and protein. These results are consistent with literature describing a positive correlation of NF45 and NF90/NF110 expression levels with cell growth and proliferation in ESCs [30, 31] and diverse cancers [32–37].

The dynamic and reciprocal chromatin association by NF90/NF110 and NF45 upon cell stimulation represent the first experimental evidence that these proteins are capable of independent chromatin interactions, in contrast to frequent biochemical characterizations of NF90/NF110 and NF45 as a heterodimer in solution [21]. The heterodimerization between NF90/NF110 and NF45 is mediated through their shared DZF domains [20], and this interaction may be regulated by cell signaling to recruit NF90/NF110 to the NF45 molecules pre-associated at the proximal promoters of IEGs.

The presence of NF45 pre-existing at the proximal promoters of inducible IEGs in nonstimulated cells identify it as a potential pioneer transcription factor [49]. These NF45 molecules may represent targets of signaling initiated at the plasma membrane, such as phorbol ester activation of protein kinase C (PKC) and downstream phosphorylation cascades. Previous proteomics studies have identified NF45 to be diversely modified, including mono- and di-methylation of the N-terminal arginine/glycine/glycine (RGG) domain, as well as phosphorylation, acetylation, and ubiquitination. The RGG domain in Xenopus ILF3 (Xilf3) has previously been shown to bind nucleic acids and methylation within this RGG domain reduced DNA-binding without affecting RNA-binding [22]. NF110, a splice variant of NF90/NF110 that also heterodimerizes with NF45 to form a NF110-NF45 complex, has been shown to be a substrate and regulator of Protein-arginine methyltransferase I (PRMT1) in mammalian cells [50]. Reduction in chromatin association of NF45 with IEG proximal promoters upon cell stimulation may be a consequence of reduced affinity for DNA following post-translational modification.

NF45 and NF90/NF110 have been shown to contribute to RNA splicing [45, 51], stabilization, and nuclear export [52, 53] and translational regulation [54, 55]. Our results here, together with previous ChIP-seq studies of NF90/NF110 [41], suggest that NF45 and NF90/110 are recruited to chromatin and contribute to transcriptional activation. The lack of consensus DNA-binding domains within these proteins indicate they are targeted to chromatin through alternative mechanisms that may involve the versatile and regulable nucleic-acid binding RGG domain, or through the dsRNA-binding domains on NF90/NF110. YY1 is a transcription factor capable of binding both DNA and RNA that has been recently shown to be retained near active promoters through its interaction with nascently transcribed RNAs [56]. Genome-wide mapping of RNA:DNA hybrids have suggested their potential function in transcriptional regulation, and NF45/*ILF2* and NF90/NF110/*ILF3* are enriched at chromatin with RNA:DNA hybrids [57].

In this study, we demonstrate that RNAi-mediated knockdown of either NF45 or NF90/NF110 attenuated inducible transcription of immediate early transcription factors, *EGR1*, *FOS* and *JUN*. NF45 and NF90/NF110 regulate gene expression through specific chromatin associations, transcriptional activation, RNA splicing, export, stabilization and translation. NF45 and NF90/NF110 therefore represent novel therapeutic targets to modulate cell responses ranging from acute inflammation to malignant proliferation.

## Supporting information

S1 Table Specific genes in immediate early (IE), delayed primary response (D-PRG) and secondary response (SRG) groups and genomic coordinates. (XLS)
